# Corrections to: Peripheral vision and pattern recognition: A review

**DOI:** 10.1167/jov.24.4.15

**Published:** 2024-04-17

##  

***CORRECTIONS TO:*** Strasburger, H., Rentschler, I., & Jüttner, M. (2011). Peripheral vision and pattern recognition: A review. *Journal of Vision*, *11*(5):13, 1–84, https://doi.org/10.1167/11.5.13.

It is a single number for which our 80-page review for peripheral vision has been repeatedly cited: the lateral horizontal extent of the visual field. We said it is about 90° of visual angle from the point of fixation. This number is wrong—the actual extent is much larger. According to the classic text by Traquair (1927), the lateral extent in each eye is around 107° of visual angle (and the horizontal extent of the entire field, from left to right, is twice that number).

The error occurs at two locations in the paper and at a potentially misleading third. In the Introduction, we stated that the horizontal diameter was “nearly” 180° (p. 3)—that is, twice 90°. Second, our comparison of visual fields of recognition and detection (Figure 14, p. 25, taken from Strasburger & Rentschler, 1996) uses 180° as the outer delimiter for the (dashed) detection field, suggesting that detection cannot occur beyond that limit. Third, in the perimetric data of Harvey and Pöppel (1972) shown in Figure 8A, the outer isopter goes to around 80° on the lateral side, which seems to suggest a limit of 90°.

How could this gross error arise? We were not alone with this misconception; it is widespread in the scientific literature and in textbooks (for a review, see Strasburger, 2020). Curiously, although a correct estimate has been known since Purkinje (1825), (cited after Wade, 1998), and is even seen in a drawing from Leonardo da Vinci (da Vinci's Manuscript D from ∼1514; Wade, personal communication), the rather limited estimate of 90° arose during the 1980s. A likely reason is the widespread use of cupola perimeters, in conjunction with the influential operational definition of the visual field by Aulhorn and Harms (1972, p. 103) as “the field of functional capacity obtained and recorded by means of perimetry.” In this view, the term “visual field” does not refer to the maximum field visible but rather to the sensitivity profile, obtained for ophthalmic diagnostic reasons, within these perimetric limits. This difference in meaning has been overlooked by those relying on the 90° border seen in standard perimetric maps.

The following changes have been made to the text in the article online:

In the last paragraph of Chapter 1, “In perimetry, one might refer to the *central visual field* with 60° diameter. *Peripheral vision* would then occur within the area from 60° up to nearly 180° horizontal diameter” has been changed to “In perimetry, one might refer to the *central visual field* with 60° diameter (30° radius). *Peripheral vision* would then occur within the area from 60° (i.e., ±30°) up to around 214° horizontal diameter.”

In the Figure 8 legend, “Characterization of the visual field by Pöppel and Harvey. (a) Perimetry data by Harvey and Pöppel (1972), i.e. light increment thresholds. Reproduced with permission from The American Academy of Optometry 1972.” has been changed to “Characterization of the visual field by Pöppel and Harvey. (a) Perimetry data by Harvey and Pöppel (1972), i.e. light increment thresholds. Reproduced with permission from The American Academy of Optometry 1972. Note that on the temporal side the visual field extends further out than seen by the outer isopter, to around 107°; it is limited here by the test spot used.”

The following has been added to the end of the Figure 14 legend: “Note that the dashed line does not represent the full visual field of detection since a small test spot is used for the perimetric data; the full field would extend to around ±107°.”

## References

[bib1] Aulhorn, E., & Harms, H. (1972). Visual perimetry. In D. Jameson & L. M. Hurvich (Eds.), *Handbook of sensory physiology, Vol. 7/4: Visual psychophysics* (pp. 102–145). Berlin: Springer.

[bib2] Harvey, L. O., Jr., & Pöppel, E. (1972). Contrast sensitivity of the human retina. *American Journal of Optometry and Archives of the American Academy of Optometry,* 49(9), 748–753. 10.1097/00006324-197209000-00007 4507460

[bib3] Purkinje, J. E. J. (1825). *Neue Beiträge zur Kenntniss des Sehens in subjectiver Hinsicht*. Berlin: G. Reimer.

[bib4] Strasburger, H. (2020). Seven myths on crowding and peripheral vision. *i-Perception,* 11(3), 1–46. 10.1177/2041669520913052 PMC7238452 32489576

[bib5] Strasburger, H., & Rentschler, I. (1996). Contrast-dependent dissociation of visual recognition and detection field. *European Journal of Neuroscience,* 8(8), 1787–1791. 8921269 10.1111/j.1460-9568.1996.tb01322.x

[bib6] Traquair, H. M. (1927). *An introduction to clinical perimetry*. London: Henry Kimpton.

[bib7] Wade, N. J. (1998). *A natural history of vision*. Cambridge, MA: MIT Press.

